# P-1985. Multi-pathogen screening of lesion swab specimens submitted for clinical testing at a national reference laboratory

**DOI:** 10.1093/ofid/ofaf695.2152

**Published:** 2026-01-11

**Authors:** Anuradha Rao, Courtney Sabino, Heather Bowers, Julie Sullivan, Leda Bassitt, Kaleb McLendon, Sharmila Talekar, Joseph Najjar, Evelyn K Williams, Nira Pollock, Carlos L Aparicio, Pamela A Miller, Eric Lai, Wilbur A Lam, Gregory L Damhorst

**Affiliations:** Emory University School of Medicine,, Atlanta, Georgia; Emory University, Atlanta, Georgia; Emory University, Atlanta, Georgia; Emory University, Atlanta, Georgia; Emory University School of Medicine, Atlanta, Georgia; Emory University, Atlanta, Georgia; Emory University/ELIAD, Atlanta, Georgia; Emory, Decatur, Georgia; Emory University School of Medicine, Atlanta, Georgia; NIH/National Institute of Bioimaging and Bioengineering (NIBIB), Boston, Massachusetts; CLAS Consulting, Miami, Florida; Echo Consulting, Excelsior, Minnesota; VentureWell, San Diego, California; Emory University School of Medicine/Georgia Institute of Technology, Atlanta, GA; Emory University, Atlanta, Georgia

## Abstract

**Background:**

Infections presenting with localized or generalized lesions have a broad range of etiologies with different treatment implications. Targeted molecular testing is not always pursued for lesion swabs but is feasible using FDA-authorized assays or laboratory-developed tests (LDTs). As part of the NIH/NIBIB Independent Test Assessment Program (ITAP) for Diagnostic Mpox Lesion Panel Test Validation, we screened de-identified remnant lesion swab specimens from a reference laboratory for the primary purpose of identifying negative specimen matrix for test validation activities. This screening provided insight into the pathogens present in lesion specimens that tested negative on the originally ordered clinical test.

**Table 1.**
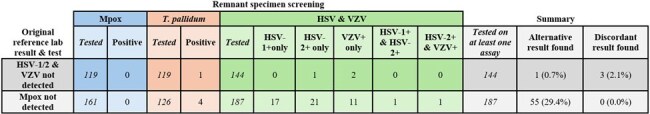
Summary of screening of remnant lesion swabs specimens performed for our test validation program. Specimens were sourced from a national reference laboratory where they had tested negative on a molecular test for Mpox or a multiplex molecular Herpes Simplex Virus (HSV)–1&2 and Varicella Zoster Virus (VZV) test as part of clinical care.

**Methods:**

346 specimens (Copan swabs in Universal Transport Media (UTM)) in which Herpes Simplex Virus (HSV)–1&2 and Varicella Zoster Virus (VZV) or Mpox were not detected at the source reference laboratory were screened for our program with at least one assay. Most specimens were further diluted 1:10 in transport media prior to testing on one or more of the following: the Xpert® Mpox assay (GeneXpert), a *Treponema pallidum* quantitative PCR LDT, and the Simplexa® HSV 1&2 Direct and VZV Swab Direct kits (LIAISON® MDX). Specimens producing positive results on any assay were not tested further, and 15 specimens with invalid results were excluded from further testing and analysis.

**Results:**

For specimens originally negative for HSV and VZV, 3 discordant results (1 HSV-2+, 2 VZV+), no Mpox, and 1 *T. pallidum* were detected (Table 1). For specimens originally negative for Mpox, 17 HSV-1+, 21 HSV-2+, 11 VZV+, 1 specimen HSV-1+ and HSV-2+, 1 specimen HSV-2+ and VZV+, 4 *T. pallidum*, and no Mpox were detected.

**Conclusion:**

Although limited by dilution procedures and lack of clinical information, our data suggest that specimens primarily tested for HSV-1/2 and/or VZV rarely exhibit *T. pallidum* or Mpox as an alternative etiology. In contrast, more than one quarter of specimens originally tested for Mpox and found to be negative were from lesions potentially attributable to *T. pallidum,* HSV, or varicella infections. In part due to the emergence of Mpox as a sexually transmitted infection in the United States, multiplex NAATs for microbial causes of lesions may be clinically useful.

**Disclosures:**

Evelyn K. Williams, PhD, Diasorin: Grant/Research Support Wilbur A. Lam, MD, PhD, Sanguina, Inc: Board Member|Sanguina, Inc: several patents that have come from my lab|Sanguina, Inc: Stocks/Bonds (Private Company)

